# Supplementary material for: Bones or Stones: How Can We Apply Geophysical Techniques in Bone Research?
Source: Int J Mol Sci. 2024 Oct 5;25(19):10733. doi: 10.3390/ijms251910733 (PMC11477212; doi:10.3390/ijms251910733)
Supplement: Supplementary file 1 [file ijms-25-10733-s001.zip › Table S1-Chemistry.pdf]

Supplementary Table S1. Correlations between chemistry parameters with others

	Chemistry parameters								
	**CaO**	**Fe_2O_3**	**K_2O**	**MgO**	**Na_2O**	**P_2O_5**	**SiO_2**	**SO_3**	**SrO**
**XRD**									
**Thermogravimetric analysis**									
H_2O	R=0.674 p=0.008				R=0.612 p=0.020	R=0.726 p=0.003			
simple organic content	R=0.778 p=0.001		R=-0.749 p=0.002		R=0.625 p=0.017	R=0.789 p=0.001	R=-0.553 p=0.040	R=0.540 p=0.045	R=0.625 p=0.017
composite organic content	R=0.838 p<0.001		R=-0.768 p=0.001			R=0.825 p<0.001	R=-0.551 p=0.041	R=0.630 p=0.016	R=0.604 p=0.022
total volatile content	R=0.810 p<0.001		R=-0.740 p=0.002		R=0.616 p=0.019	R=0.823 p<0.001		R=0.559 p=0.038	R=0.606 R=0.022
**FTIR ATR**									
H_2O+CH			R=-0.583 p=0.029						
CH	R=0.683 p=0.007		R=-0.809 p<0.001			R=0.798 p=0.001	R=-0.586 p=0.028		
PO_4+CO_3	R=0.766 p=0.001	R=-0.713 p=0.004	R=-0.832 p<0.001	R=0.608 p=0.021		R=0.729 p=0.003	R=-0.728 p=0.003		
CO_3	R=0.784 p=0.001	R=-0.639 p=0.014	R=-0.857 p<0.001			R=0.805 p=0.001	R=-0.741 p=0.002		R=0.601 p=0.023
amide I+CO_3	R=0.695 p=0.006		R=-0.844 p<0.001			R=0.747 p=0.002	R=-0.676 p=0.008	R=0.565 p=0.035	
amide I	R=0.777 p=0.001		R=-0.672 p=0.008			R=0.622 p=0.017	R=-0.559 p=0.038	R=0.567 p=0.035	
CO_3 / (PO_4+CO_3)	R=0.705 p=0.005		R=-0.784 p=0.001		R=0.581 p=0.030	R=0.762 p=0.002	R=-0.686 p=0.007		R=0.728 p=0.003
**ICP-OES**									
Cu		R=0.635 p=0.015	R=0.673 p=0.008				R=0.541 p=0.046		
**ICP-MS**									
Ba	R=0.570				R=0.642	R=0.644			R=0.798

	p=0.033				p=0.013	p=0.013			p=0.001
Ce		R=0.559<br>p=0.038							
Co	R=0.794<br>p=0.001		R=-0.637<br>p=0.014		R=0.574<br>p=0.032	R=0.715<br>p=0.004			R=0.645<br>p=0.013
Cr				R=-0.708<br>p=0.005					
La		R=0.669<br>p=0.009	R=0.660<br>p=0.010				R=0.696<br>p=0.006		
Mo				R=0.692<br>p=0.006	R=0.603<br>p=0.022				
Ni		R=0.541<br>p=0.046	R=0.562<br>p=0.037	R=-0.708<br>p=0.005				R=-0.544<br>p=0.044	
Rb		R=0.642<br>p=0.013	R=0.615<br>p=0.019				R=0.601<br>p=0.023		
Sb				R=-0.566<br>p=0.035					
Sn			R=-0.563<br>p=0.036					R=0.684<br>p=0.007	
Sr					R=0.757<br>p=0.002	R=0.538<br>p=0.047			R=0.901<br>p<0.001
**Chemistry**									
CaO			R=-0.681<br>p=0.007		R=0.580<br>p=0.030	R=0.824<br>p<0.001	R=-0.608<br>p=0.021		
Fe_2O_3			R=0.720<br>p=0.004				R=0.900<br>p<0.001		
K_2O	R=-0.681<br>p=0.007	R=0.720<br>p=0.004				R=-0.702<br>p=0.005	R=0.771<br>p=0.001		
Na_2O	R=0.580<br>p=0.030								R=0.538<br>p=0.047
P_2O_5	R=0.824<br>p<0.001		R=-0.702<br>p=0.005						
SiO_2	R=-0.608<br>p=0.021	R=0.900<br>p<0.001	R=0.771<br>p=0.001						
SO_3									
SrO					R=0.538				

					p=0.047				
