# Supplementary material for: Bones or Stones: How Can We Apply Geophysical Techniques in Bone Research?
Source: Int J Mol Sci. 2024 Oct 5;25(19):10733. doi: 10.3390/ijms251910733 (PMC11477212; doi:10.3390/ijms251910733)
Supplement: Supplementary file 1 [file ijms-25-10733-s001.zip › Table S2-ICP-OES.pdf]

Supplementary Table S2. Correlations between ICP-OES parameters with others

	ICP-OES parameters		
	**Cu**	**Li**	**Zn**
**XRD**			
degree of cristallinity	R=0.846<br>p<0.01		
**Thermogravimetric analysis**			
H_2O			
simple organic content	R=-0.670<br>p=0.009		
composite organic content	R=-0.619<br>p=0.018		
total volatile content	R=-0.622<br>p=0.018		
**FTIR-ATR**			
PO_4+CO_3	R=-0.655<br>p=0.011		
CO_3	R=-0.598<br>p=0.024		
**ICP-MS**			
Co	R=-0.698<br>p=0.006		
La	R=0.581<br>p=0.029		
Mn			R=0.712<br>p=0.004
Mo			R=0.733<br>p=0.003
Ni		R=0.653<br>p=0.011	
Rb	R=0.591<br>p=0.026		
Sb	R=0.540<br>p=0.046	R=0.680<br>p=0.008	
Sn	R=-0.713<br>p=0.004		
Sr			R=0.571<br>p=0.033
Zn			R=1.000<br>p=0
**Chemistry**			
Fe_2O_3	R=0.635<br>p=0.015		
K_2O	R=0.673<br>p=0.008		
SiO_2	R=0.541<br>p=0.046		
