# Supplementary material for: Bones or Stones: How Can We Apply Geophysical Techniques in Bone Research?
Source: Int J Mol Sci. 2024 Oct 5;25(19):10733. doi: 10.3390/ijms251910733 (PMC11477212; doi:10.3390/ijms251910733)
Supplement: Supplementary file 1 [file ijms-25-10733-s001.zip › Table S3-ICP-MS.pdf]

Supplementary Table S3. Correlations between ICP-MS parameters with others

	ICP-MS parameters														
	**Ba**	**Ce**	**Co**	**Cr**	**Cu**	**La**	**Mn**	**Mo**	**Ni**	**Pb**	**Rb**	**Sb**	**Sn**	**Sr**	**Zn**
**XRD**															
apatite Hb										R=-0.593<br>p=0.025					
degree of cristallinity			R=-0.604<br>p=0.022								R=0.599<br>p=0.038		R=-0.574<br>p=0.032		
**Thermogravimetric analysis**															
H_2O	R=0.612<br>p=0.020		R=0.745<br>p=0.002						R=-0.560<br>p=0.037			R=-0.547<br>p=0.043		R=0.626<br>p=0.017	
simple organic content	R=0.766<br>p=0.001		R=0.887<br>p<0.001			R=-0.606<br>p=0.022			R=-0.567<br>p=0.034			R=-0.541<br>p=0.046	R=0.664<br>p=0.010	R=0.702<br>p=0.005	
composite organic content	R=0.715<br>p=0.004		R=0.887<br>p<0.001						R=-0.557<br>p=0.039				R=0.725<br>p=0.003	R=0.582<br>p=0.029	
CO_3		R=0.621<br>p=0.018				R=-0.737<br>p=0.003					R=0.676<br>p=0.008				
total volatile content	R=0.748<br>p=0.002		R=0.903<br>p<0.001						R=-0.614<br>p=0.019				R=0.669<br>p=0.009	R=0.670<br>p=0.009	
**FTIR ATR**															
CH			R=0.558<br>p=0.038												
PO_4+CO_3			R=0.801<br>p=0.001			R=-0.575<br>p=0.032			R=-0.646<br>p=0.013				R=0.598<br>p=0.024		
CO_3	R=0.624<br>p=0.017		R=0.808<br>p<0.001			R=-0.631<br>p=0.016			R=-0.557<br>p=0.039				R=0.612<br>p=0.020	R=0.616<br>p=0.019	
amide I+CO_3			R=0.591<br>p=0.026			R=-0.537<br>p=0.048							R=0.578<br>p=0.031		
amide I			R=0.570<br>p=0.033										R=0.576<br>p=0.031		
CO_3 / (PO_4+CO_3)	R=0.720<br>p=0.004		R=0.727<br>p/0.003			R=-0.621<br>p=0.018							R=0.553<br>p=0.040	R=0.716<br>p=0.004	
**ICP-OES**															
Cu			R=-0.698<br>p=0.006			R=0.581<br>p=0.029					R=0.591<br>p=0.026	R=0.540<br>p=0.046	R=-0.713<br>p=0.004		
Li									R=0.653<br>p=0.011			R=0.680<br>p=0.008			
Zn							R=0.712<br>p=0.004	R=0.733<br>p=0.003						R=0.571<br>p=0.033	R=1.000<br>p=0
**ICP-MS**															
Ba			R=0.786<br>p=0.001											R=0.852<br>p<0.001	R=0.717<br>p=0.004
Ce				R=0.649		R=0.917				R=0.570	R=0.904				

				p=0.012		p<0.001				p=0.033	p<0.001				
Co	R=0.786<br>p=0.001								R=-0.555<br>p=0.040				R=0.760<br>p=0.002	R=0.666<br>p=0.009	
Cr		R=0.649<br>p=0.012				R=0.649<br>p=0.012			R=0.664<br>p=0.010		R=0.663<br>p=0.010				
La		R=0.917<br>p<0.001		R=0.649<br>p=0.012							R=0.959<br>p<0.001				
Mn								R=0.911<br>p<0.001							
Mo							R=0.911<br>p<0.001								R=0.734<br>p=0.003
Ni			R=-0.555<br>p=0.040	R=0.664<br>p=0.010								R=0.683<br>p=0.007			
Pb		R=0.570<br>p=0.033										R=0.700<br>p=0.005			
Rb		R=0.904<br>p<0.001		R=0.663<br>p=0.010		R=0.959<br>p<0.001									
Sb									R=0.683<br>p=0.007	R=0.700<br>p=0.005					
Sn			R=0.760<br>p=0.002												
Sr	R=0.852<br>p<0.001		R=0.666<br>p=0.009												R=0.572<br>p=0.033
Zn	R=0.717<br>p=0.004							R=0.734<br>p=0.003						R=0.572<br>p=0.033	
**Chemistry**															
CaO	R=0.570<br>p=0.033		R=0.794<br>p=0.001	R=-0.708<br>p=0.005											
Fe_2O_3		R=0.559<br>p=0.038				R=0.669<br>p=0.009					R=0.642<br>p=0.013				
K_2O			R=-0.637<br>p=0.014			R=0.660<br>p=0.010			R=0.562<br>p=0.037		R=0.615<br>p=0.019		R=-0.563<br>p=0.036		
MgO				R=-0.708<br>p=0.005				R=0.692<br>p=0.006				R=-0.566<br>p=0.035			
Na_2O	R=0.642<br>p=0.013		R=0.574<br>p=0.032					R=0.603<br>p=0.022						R=0.751<br>p=0.002	
P_2O_5	R=0.644<br>p=0.013		R=0.715<br>p=0.004											R=0.538<br>p=0.047	
SiO_2						R=0.696<br>p=0.006					R=0.601<br>p=0.023				
SO_3									R=-0.544<br>p=0.044				R=0.684<br>p=0.007		
SrO	R=0.798<br>p=0.001	R=0.645<br>p=0.013												R=0.901<br>p<0.001	
