# Supplementary material for: Bones or Stones: How Can We Apply Geophysical Techniques in Bone Research?
Source: Int J Mol Sci. 2024 Oct 5;25(19):10733. doi: 10.3390/ijms251910733 (PMC11477212; doi:10.3390/ijms251910733)
Supplement: Supplementary file 1 [file ijms-25-10733-s001.zip › Table S4-XRD.pdf]

Supplementary Table S4. Correlations between XRD parameters with others

	**XRD parameters**				
	**apatite peak intensity (AU)**	**ratio of peak intensity at (211) and (222)**	**ratio of peak intensity at (211) and (300)**	**degree of cristallinity**	**C-axis**
**Thermogravimetric analysis**					
simple organic content				R=-0.541 p=0.046	
CO_3			R=-0.567 p=0.034		R=-0.591 p=0.026
**ICP-OES**					
Cu				R=0.846 p<0.001	
**ICP-MS**					
Co				R=-0.604 p=0.022	
Mn					R=-0.574 p=0.032
Pb	R=-0.593 p=0.025				
Rb				R=0.599 p=0.038	
Sn				R=-0.574 p=0.032	
