# Supplementary material for: Bones or Stones: How Can We Apply Geophysical Techniques in Bone Research?
Source: Int J Mol Sci. 2024 Oct 5;25(19):10733. doi: 10.3390/ijms251910733 (PMC11477212; doi:10.3390/ijms251910733)
Supplement: Supplementary file 1 [file ijms-25-10733-s001.zip › Table S5-TGA.pdf]

Supplementary Table S5. Correlations between thermogravimetric analysis parameters with others

	Thermogravimetric analysis parameters				
	**H_2O**	**simple organic content**	**composite organic content**	**CO_3 content**	**total volatile content**
**XRD**					
ratio of 211 and 300 peak intensity				R=-0.567 p=0.034	
degree of cristallinity		R=-0.541 p=0.046			
C-axis				R=-0.591 p=0.026	
**FTIR ATR**					
CH	R=0.654 p=0.011	R=0.669 p=0.009	R=0.784 p=0.001		R=0.732 p=0.003
PO_4+CO_3	R=0.777 p=0.001	R=0.818 p<0.001			R=0.833 p<0.001
CO_3	R=0.778 p=0.001	R=0.907 p<0.001			R=0.907 p<0.001
amide I+CO_3	R=0.557 p=0.039	R=0.780 p=0.001			R=0.716 p=0.004
amide I	R=0.599 p=0.024	R=0.731 p=0.003			R=0.673 p=0.008
CO_3 / (PO_4+CO_3)	R=0.667 p=0.009	R=0.882 p<0.001	R=0.874 p<0.001		R=0.862 p<0.001
**ICP-OES**					
Cu		R=-0.670 p=0.009	R=-0.619 p=0.018		R=-0.622 p=0.018
**ICP-MS**					
Ba	R=0.612 p=0.020	R=0.766 p=0.001	R=0.715 p=0.004		R=0.748 p=0.002
Ce		R=-0.535 p=0.049		R=0.621 p=0.018	
Co	R=0.745 p=0.002	R=0.887 p<0.001	R=0.887 p<0.001		R=0.903 p<0.001
La		R=-0.606 p=0.022		R=-0.737 p=0.003	
Ni	R=-0.560 p=0.037	R=-0.567 p=0.034	R=-0.557 p=0.039		R=-0.614 p=0.019
Rb				R=0.676 p=0.008	
Sb	R=-0.547 p=0.043	R=-0.541 p=0.046			
Sn		R=0.664 p=0.010	R=0.725 p=0.003		R=0.669 p=0.009
Sr	R=0.626 p=0.017	R=0.702 p=0.005	R=0.582 p=0.029		R=0.670 p=0.009
**Chemistry**					
CaO	R=0.674 p=0.008	R=0.778 p=0.001	R=0.838 p<0.01		R=0.810 p<0.001
K_2O		R=-0.749 p=0.002	R=-0.768 p=0.001		R=-0.740 p=0.002
Na_2O	R=0.612 p=0.020	R=0.625 p=0.017			R=0.616 p=0.019
P_2O_5	R=0.726 p=0.003	R=0.789 p=0.001	R=0.825 p<0.001		R=0.823 p<0.001
SO_3		R=0.540 p=0.045	R=0.630 p=0.016		R=0.559 p=0.038
SiO_2		R=-0.553 p=0.040	R=-0.551 p=0.041		
SrO		R=0.625 p=0.017	R=0.604 p=0.021		R=0.606 p=0.022
