# Supplementary material for: Bones or Stones: How Can We Apply Geophysical Techniques in Bone Research?
Source: Int J Mol Sci. 2024 Oct 5;25(19):10733. doi: 10.3390/ijms251910733 (PMC11477212; doi:10.3390/ijms251910733)
Supplement: Supplementary file 1 [file ijms-25-10733-s001.zip › Table S6-FTIR-ATR.pdf]

Supplementary Table S6. Correlations between FTIR-ATR parameters with others

	FTIR ATR parameters							
	H_2O+CH	CH	PO_4+CO_3	CO_3	amide I +CO_3	amide I	amide I / (PO_4+CO_3)	CO_3 / (PO_4+CO_3)
**Thermogravimetric analysis**								
H_2O		R=0.654<br>p=0.011	R=0.777<br>p=0.001	R=0.778<br>p=0.001	R=0.557<br>p=0.039	R=0.599<br>p=0.024		R=0.667<br>p=0.009
simple organic content		R=0.669<br>p=0.009	R=0.818<br>p<0.001	R=0.907<br>p<0.001	R=0.780<br>p=0.001	R=0.731<br>p=0.003		R=0.882<br>p<0.001
composite organic content		R=0.784<br>p=0.001						R=0.874<br>p<0.001
total volatile content		R=0.732<br>p=0.003	R=0.833<br>p<0.001	R=0.907<br>p<0.001	R=0.716<br>p=0.004	R=0.670<br>p=0.008		R=0.862<br>p<0.001
**ICP-OES**								
Cu			R=-0.655<br>p=0.011	R=-0.598<br>p=0.024				
**ICP-MS**								
Ba				R=0.624<br>p=0.017				R=0.720<br>p=0.003
Co		R=0.558<br>p=0.038	R=0.801<br>p=0.001	R=0.808<br>p<0.001	R=0.591<br>p=0.026	R=0.570<br>p=0.033		R=0.727<br>p=0.003
La			R=-0.575<br>p=0.032	R=-0.631<br>p=0.016	R=-0.537<br>p=0.048			R=-0.621<br>p=0.018
Ni			R=-0.646<br>p=0.013	R=-0.557<br>p=0.039				
Sn			R=0.598<br>p=0.024	R=0.612<br>p=0.020	R=0.578<br>p=0.031	R=0.576<br>p=0.031		R=0.553<br>p=0.040
Sr				R=0.616<br>p=0.019				R=0.716<br>p=0.004
**Chemistry**								
CaO		R=0.683<br>p=0.007	R=0.766<br>p=0.001	R=0.784<br>p=0.001	R=0.695<br>p=0.006	R=0.777<br>p=0.001		R=0.705<br>p=0.005
Fe_2O_3			R=-0.713<br>p=0.004	R=-0.639<br>p=0.014				
K_2O	R=-0.583<br>p=0.029	R=-0.809<br>p<0.001	R=-0.832<br>p<0.001	R=-0.857<br>p<0.001	R=-0.844<br>p<0.001	R=-0.672<br>p=0.008		R=-0.784<br>p=0.001
MgO			R=0.608<br>p=0.021					
Na_2O								R=0.581<br>p=0.030
P_2O_5		R=0.798<br>p=0.001	R=0.729<br>p=0.003	R=0.805<br>p=0.001	R=0.747<br>p=0.002	R=0.622<br>p=0.017		R=0.762<br>p=0.002
SiO_2		R=-0.586<br>p=0.028	R=-0.728<br>p=0.003	R=-0.741<br>p=0.002	R=-0.676<br>p=0.008	R=-0.559<br>p=0.038		R=-0.686<br>p=0.007
SO_3					R=0.565<br>p=0.035	R=0.567<br>p=0.035		
SrO				R=0.601<br>p=0.023				R=0.728<br>p=0.003
